# Patient with Inflammatory Bowel Disease in a Dental Office—Which Antibiotic to Choose?—Narrative Review

**DOI:** 10.3390/jcm14238392

**Published:** 2025-11-26

**Authors:** Stanisław Niemczyk, Wojciech Niemczyk, Katarzyna Bąk-Drabik, Katarzyna Latusek-Kotyczka, Anna Zawilska, Rafał Wiench, Jakub Hadzik, Marzena Dominiak

**Affiliations:** 1 Municipal Hospital No. 4 in Gliwice, Zygmunta Starego 20, 44-100 Gliwice, Poland; stanislaw.niemczyk.research@gmail.com; 2 Department of Dental Surgery, Faculty of Dentistry, Wroclaw Medical University, Krakowska 26, 50-425 Wroclaw, Poland; 3 Department of Periodontal Diseases and Oral Mucosa Diseases, Faculty of Medical Sciences in Zabrze, Medical University of Silesia, pl. Traugutta 2, 41-800 Zabrze, Poland; 4 Department of Pediatrics, School of Medicine with the Division of Dentistry in Zabrze, Medical University of Silesia, 40-752 Katowice, Poland; 5 Department of Conservative Dentistry with Endodontics, Faculty of Medical Sciences in Zabrze, Medical University of Silesia, PlacAkademicki 17, 41-902 Bytom, Poland

**Keywords:** antibiotic prohylaxis, dentistry, inflammatory bowel diseases

## Abstract

**Background/Objectives**: The rising global prevalence of inflammatory bowel disease (IBD), encompassing Crohn’s disease and ulcerative colitis, has resulted in an increase in the number of affected patients requiring dental care. The heightened risk of *Clostridioides difficile* infection (CDI) in IBD patients, particularly when exposed to commonly used dental antibiotics, is attributable to their altered gut microbiota and frequent immunosuppressive therapy. The objective of this review is to evaluate current antibiotic strategies for dental management in IBD and to identify safe and effective alternatives that minimise CDI risk. **Methods**: A narrative review was conducted in accordance with the SANRA guidelines. A comprehensive analysis of literature sourced from PubMed, Embase, Scopus, and Google Scholar was conducted. **Results**: The available evidence suggests that first- and second-line dental antibiotics—amoxicillin, ampicillin, and clindamycin—carry the highest risk of CDI. In contrast, metronidazole, which exhibits a comparable antimicrobial spectrum, has been shown to possess significantly reduced CDI potential and minimal disruption of gut microbiota. The utilisation of emerging local delivery systems, such as platelet-rich fibrin (PRF), has the potential to further reduce systemic antibiotic exposure. The adjunctive use of probiotics, prebiotics and synbiotics has been demonstrated to have the capacity to maintain microbial balance during therapy. **Conclusions**: Tailored, microbiome-conscious antibiotic strategies are essential in dental management of IBD patients. Further clinical research is needed to develop evidence-based guidelines and validate promising adjunctive approaches.

## 1. Introduction

Inflammatory Bowel Disease (IBD) is a group of disorders that include Crohn’s Disease (CD) and Ulcerative Colitis (UC). These are complex, multifactorial autoimmune diseases characterised by chronic inflammation of the gastrointestinal tract caused by a disturbed structure of the mucosa and an altered composition of intestinal microorganisms. CD and UC are differentiated based on clinical symptoms and the location of histopathological changes [1,2]. All over the world, there is a continuous increase in the incidence of these diseases [3,4,5,6]. The incidence of IBD has traditionally been highest in North America and Western Europe, with many cohorts suggesting a substantial secular increase over the second half of the twentieth century. However, the incidence of IBD is also increasing in emerging populations such as Asia, suggesting that changing environmental factors play a significant role in disease pathogenesis. Lifestyle, diet, and behaviour, as well as perturbations of the gut microbiota through antibiotic therapy, may also have important roles in disease pathogenesis [7].

The common clinical features ofUC include abdominal pain, diarrhoea, rectal bleeding and other gastrointestinal symptoms. In addition to these prevalent symptoms of the gastrointestinal tract, 6–40% of patients suspected of having IBD may also manifest various extraintestinal manifestations. It is important to note that extraintestinal manifestations may occur prior to the definitive diagnosis of IBD [8]. It has been established that up to 9% of patients diagnosed with IBD also exhibit oral manifestations [9].

*Clostridioides difficile* infection (CDI) has been increasing in frequency and severity in patients with inflammatory bowel disease. The potential for progression from colonisation to symptomatic CDI is contingent upon exposure to risk factors, including but not limited to contact with the healthcare environment, antibiotic use, and advanced age [10]. The incidence of CDI in patients with IBD is 32.9 per 1000 patients, while in patients with no IBD, the incidence of CDI was 9.3 per 1000 [11]. Studies have shown worse clinical outcomes in patients with concomitant CDI and IBD [12]. In patients with IBD, CDI is associated with increased morbidity, including escalation in IBD medical therapy, urgent colectomy, increased hospitalisation, and excess mortality [13]. The exacerbation of diarrhoea resulting from an underlying flare-up of IBD can frequently be clinically indistinguishable from the presence of superimposed CDI. The management of an IBD flare entails the administration of immunosuppressive therapy, whereas the management of CDI involves the administration of antibiotics. The infection itself is a contributing factor to IBD flare management, which renders the process of treatment challenging [14].

Disturbingly, the first- and second-line antibiotics used in dentistry are among the group of antibiotics with the highest risk of CDI [15,16]. Despite this, current dental guidelines do not provide specific recommendations for antibiotic use in patients with IBD, treating them instead as part of a broader “non-immunocompromised” category, which does not reflect their unique risk profile. Therefore, the aim of this study was to review current recommendations and clinical evidence on antibiotic therapy in dentistry and to assess their applicability to patients with inflammatory bowel disease. In particular, this review sought to determine which antibiotics can be used safely and effectively in this population while minimising the risk of Clostridioides difficile infection and preserving gut microbiota stability. The important goal of this review is to highlight the lack of appropriate guidelines and shed light on the direction of future research, emphasising the significance of the problem.

## 2. Materials and Methods

Due to the nature of the issue, the authors chose to do a narrative review, but in ensuring its reliability, they followed the Scale for the Assessment of Narrative Review Articles (SANRA) guidelines [17].

### 2.1. Search Strategy

The PubMed, Google Scholar, Embase and Scopus databases were searched for articles published from inception to October 2025 in English and Polish using keywords such as (“Dentistry” OR “Oral health” OR “Stomatology”) AND (“CDI” OR “Clostridium difficile” OR “Clostridioides difficile” OR “periprocedural antibiotic therapy” OR “antibiotic therapy”) AND (“IBD” OR “Crohn disease” OR “inflammatory bowel disease” OR “Ulcerative Colitis”). The abstracts resulting from the searches underwent aninitial review to see whether they fit with the search requirements. Articles were selected based upon their relevancy to general medicine, prioritising original works, clinical trials, and clinical practice guidelines based upon the quality ofthe journal and the authors’ experience in the area. Additionally, a forward/backward search strategy was performed. The exact search phrases for specific databases, along with the number of results obtained, are presented in Table 1.

### 2.2. Inclusion and Exclusion Criteria

The selection of studies was conducted in accordance with a predetermined set of inclusion and exclusion criteria, which were established prior to the commencement of the review process. Eligible publications included original research articles, clinical trials, observational studies, systematic reviews, meta-analyses, and relevant narrative reviews that addressed antibiotic therapy in dentistry, oral health considerations in IBD, the risk of CDI, or microbiome-related safety concerns associated with dental antibiotics. The present study has drawn upon a range of research reports that have documented data on adult and paediatric patients diagnosed with Crohn’s disease, ulcerative colitis, or indeterminate colitis. The corpus of articles selected for inclusion encompassed those delineating microbiological profiles of odontogenic infections, the pharmacology of antibiotics employed in dental practice, and adjunctive microbiome-supportive therapies (e.g., probiotics, local antibiotic delivery systems). These were included if they were deemed to be pertinent to clinical decision-making in the context of dental care for patients with IBD. Publications in English or Polish were eligible for inclusion in the review due to the reviewers’ proficient command of the respective languages.

Exclusion criteria encompassed studies not related to dental management, antibiotics, or IBD; papers lacking clinical relevance (e.g., animal studies without translational applicability); articles exclusively focusing on gastrointestinal therapy without any reference to dental implications; and studies assessing antibiotics not used in dentistry. Case reports, conference abstracts, correspondence, and non-peer-reviewed materials were excluded unless they provided clinically meaningful insights not available elsewhere. Duplicate publications or studies with insufficient methodological detail were removed. In instances where multiple papers reported overlapping data from the same cohort, the most comprehensive or recent publication was included. Following the removal of duplicates, 1112 unique records remained for screening.

### 2.3. Bias Minimization

In order to enhance the methodological rigour and reduce the risk of bias, several measures were implemented throughout the search and selection process. Firstly, four major databases (i.e., PubMed, Embase, Scopus and Google Scholar) were utilisedin order to ensure comprehensive coverage and to minimise database-specific bias. Search terms and eligibility criteria were defined a priori in order to prevent subjective selection during screening.

Secondly, all titles, abstracts and full texts were screened independently by two reviewers. Discrepancies were resolved through discussion, thereby minimising selection bias and increasing consistency. In order to ensure accurate interpretation whilst maintaining broad international representation, language restrictions were limited to English and Polish, the respective languages in which the reviewers were fluent. In order to circumvent the potential for duplication bias, a meticulous examination of the overlapping datasets was undertaken; in such instances, the most recent or comprehensive publication was selected for inclusion.

Thirdly, the focus was exclusively on peer-reviewed literature and high-quality sources, encompassing original studies, clinical trials, systematic reviews, and meta-analyses. Non-reviewed materials, conference abstracts, and anecdotal reports were excluded, unless they contributed unique, clinically relevant insights. This approach served to limit the influence of studies of a low quality or those which were methodologically unclear. Finally, emphasis was placed on transparent reporting of the screening process, the use of explicit inclusion and exclusion criteria, and the synthesis of findings to reduce interpretative and reporting bias.

## 3. Pathophysiology of IBD Relevant to Dental Practice

### 3.1. Immune Dysregulation and Microbiome Alterations

The aetiology of immune dysregulation in IBD is attributable to a bidirectional interaction between host immunity and the gut microbiota. Alterations in microbial composition are a consequence of, and a perpetuating factor in, the process of chronic intestinal inflammation. Single-cell transcriptomic profiling has been demonstrated to reveal widespread reprogramming across epithelial, stromal and immune compartments in Crohn’s disease. This reprogramming is characterised by the activation of myeloid subsets, the expansion of inflammatory fibroblasts, and the dysregulation of cytokine networks, which collectively shape a pro-inflammatory tissue environment [18].

In addition to the observed immune abnormalities, patients exhibit characteristic gut dysbiosis, characterised by a reduction in butyrate-producing Firmicutes and an expansion of Proteobacteria. This disruption of colonocyte metabolism and impairment of epithelial hypoxia results in the promotion of the growth of facultative anaerobes associated with inflammation [19].

The loss of beneficial microbial metabolites, such as short-chain fatty acids, as well as alterations to tryptophan metabolism and disrupted bile-acid processing, have been demonstrated to further weaken mucosal barrier integrity and impair regulatory immune pathways. Collectively, these alterations create a self-reinforcing loop in which microbial imbalance enhances mucosal immune activation, while immune-mediated tissue injury further reshapes the gut microbial ecosystem [20].

### 3.2. Malabsorption and Nutritional Deficiencies

Malabsorption is a common occurrence in cases of IBD, arising from a combination of factors including, but not limited to, chronic mucosal inflammation, a reduced absorptive surface, ileal involvement or resection, and disturbances in bile acid metabolism. Consequently, nutritional imbalances frequently contribute to or exacerbate oral lesions observed in IBD [21,22,23].

The pathophysiology of iron deficiency is multifactorial, arising from chronic intestinal bleeding, inflammation-driven impaired absorption, and inadequate intake. The condition is closely associated with the presence of oral signs, including angular cheilitis, atrophic glossitis, mucosal pallor, and delayed healing. The presence of iron-related oral changes in malnourished IBD patients has been well documented, and such changes frequently coexist with other micronutrient deficits [24].

Vitamin B12 deficiency has been observed to develop predominantly in cases of Crohn’s disease involving the ileum or following ileal resection and may also be associated with bacterial overgrowth [25]. B12 depletion has been demonstrated to result in the onset of glossitis, burning mouth symptoms, mucosal atrophy, and impaired epithelial regeneration. Its association with elevated genomic instability in IBD further substantiates its pertinence to oral mucosal fragility [26]. Insufficient folate has been demonstrated to be a contributing factor to the development of oral ulcers, erythematous mucosa, and impaired healing capacity, with the potential to exacerbate recurrent aphthous lesions [27,28,29].

Vitamin D deficiency is a prevalent comorbidity in patients suffering from IBD, due to a combination of factors including impaired fat absorption, reduced intake, inflammation, and limited sun exposure. A deficiency of this vitamin has been associated with an increased susceptibility to oral infections, periodontal inflammation, and impaired mucosal healing [30,31,32,33].

## 4. Oral Manifestations of IBD and Treatment

### 4.1. IBD Group Diseases and the Condition of the Oral Cavity

Oral manifestations of IBD can be specific or non-specific and can be caused by intestinal malabsorption or induced by pharmacological treatments [34]. It has been demonstrated that all drug classes employed in the treatment of IBD, including immunosuppressants and corticosteroids [35], can result in the formation of lesions within the oral cavity [24,34,36]. Additionally, individuals with inflammatory bowel disease are at an elevated risk of developing periodontitis, which can be attributed to alterations in their oral microbiota and the presence of common inflammatory processes [37,38].

**Figure 1:**
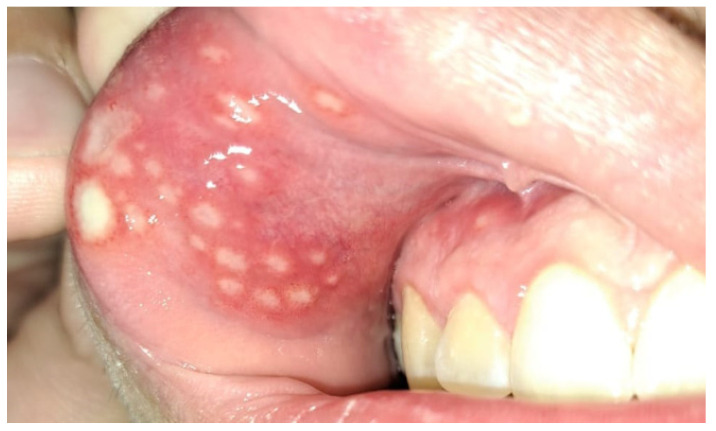
Patient aged 17 with diagnosed ulcerative colitis. Numerous aphthous-like lesions in the oral vestibule near the incisor teeth and canine, involving both the associated gingival mucosa and the mucosa of the upper lip.

**Figure 2:**
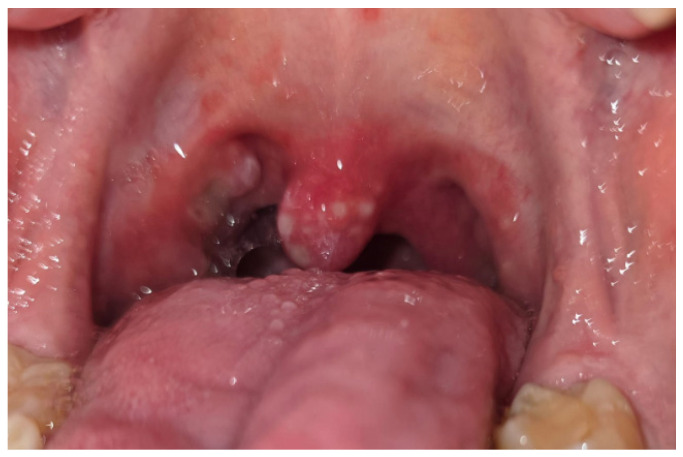
Patient aged 17 with diagnosed ulcerative colitis. Aphthous lesions in the soft palate, palatine tonsils and palatine uvula.

**Figure 3:**
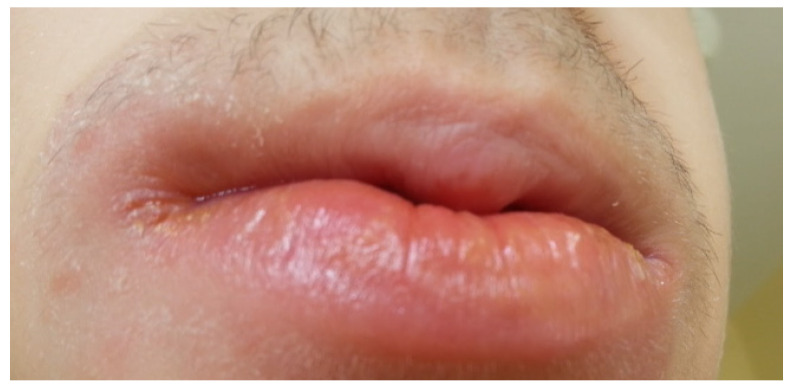
Patient 16 years old diagnosed with Crohn’s disease. Red labial inflammation with swelling.

A significant relationship has been found between the use of azathioprine and mesalazine by patients and the occurrence of oral manifestations of the disease [39]. Some of these manifestations, such as aphthae (Figure 1 and Figure 2), buccal and labial mucosal swelling (Figure 3), mucosal tags, deep linear ulcerations, and cobblestoned oral mucosa, are more indicative of CD, while pyostomatitisvegetans is correlated to UC [34,40].

The therapy for patients with IBD involves modulating the body’s inflammatory response. The drugs used are immunosuppressive and include immunosuppressants (azathioprine, mercaptopurine, cyclosporine) and corticosteroids (prednisone, budesonide) [35].

### 4.2. Treatment of the Oral Lesions

The primary guiding principle for managing oral lesions in IBD involves pinpointing their root cause. These may stem directly from IBD, malnutrition or the use of concurrent medications.

The objective in managing oral lesions among individuals with IBD is to alleviate pain, expedite lesion healing, and forestall secondary infections. Treatment approaches vary based on the underlying cause, severity of clinical manifestation, and the specific symptoms of the oral lesions. Options for treatment encompass both topical and/or systemic medications [24].

Treating particular oral lesions in CD and pyostomatitis vegetans, conditions that can arise in both diseases, consistently focuses on addressing and managing the underlying disease. Typically, lesions show positive responses to treatment for IBD [41,42]. When this outcome is not achieved, the treatment for oral manifestations mirrors the approaches used in gastroenterological care. Pharmacological options encompass topical or systemic corticosteroids, immunosuppressive agents, and biologics, particularly anti-Tumour Necrosis Factor (TNF)-α drugs. Topical treatments may involve intralesional injections, mouthwashes, and ointments, typically starting with corticosteroid ointments and/or mouthwashes, along with nonsteroidal anti-inflammatory pastes. If there is no response, intralesional corticosteroid injections may be administered. If symptoms persist, a systemic approach involving corticosteroids becomes necessary [42,43].

## 5. Dental Infection Susceptibility in IBD

The examination of purulent content collected from odontogenic foci has revealed the presence of *Streptococcus viridans*, *Peptostreptococcus*, as well as bacteria of *the Prevotella*, *Bacteroides*, and *Actinomyces species* [44]. Recent studies indicate that anaerobic bacteria are increasingly playing a significant role in odontogenic head and neck infections [45,46,47]. Compared to the healthy population in a similar age range, patients with IBD have a higher incidence of caries, and thus odontogenic inflammation, which is most often caused by it [24,34]. The caries lesions are mainly caused by limitations in optimal nutrition (low-fibre diet) [24,48,49]. This may also be due to the sometimes-used form of treatment for flare-ups of the disease in the form of a liquid diet or parenteral nutrition, which affects the lack of mechanical cleaning of the teeth from dental plaque by food [50,51,52].

## 6. Clostridioides Difficile Infection in IBD Patients

### 6.1. Why Are Patients with IBD a High-Risk Group?

*Clostridioides difficile* is a Gram-Positive anaerobic bacillus that produces endospores. It is commonly found in the digestive tract of humans, animals, and the environment [53]. It is one of the most common nosocomial infections and the leading cause of healthcare-associated diarrhoea worldwide [53,54]. The most important risk factors include: antibiotic treatment, old age, hospitalisation, or stay in a nursing home [55]. Clinical presentation can vary from asymptomatic carriage to varying degrees of diarrhoea, and even to the most severe life-threatening colitis leading to death [53,55]. It is important to minimise the risk of *Clostridioides difficile* infection as 20–30% of patients with CDI may develop recurrent infections and up to 60% of patients may develop further episodes after a first relapse [54]. Patients with IBD in complete remission who are not exposed to antibiotics or hospitalisation are much more likely to be carriers of CDI than healthy people (8.2% vs. 1.0%, respectively) [56,57]. IBD patients treated with immunomodulatory drugs and antibiotics are at a significantly higher risk of developing CDI than those treated with antibiotics alone. Shomron Ben-Horin Et Al. [58] found that 12% of patients on combination therapy developed CDI compared to no CDI patients on antibiotics alone.

### 6.2. Consequences of CDI in This Population

Studies over the years have shown that the main and most common infectious cause of exacerbations in IBD are *Clostridioides difficile* infection [59]. In addition, the long-term colectomy risk was significantly higher for IBD patients with CDI compared to those without CDI (odds ratio [OR]: 2.22, 95% confidence interval [CI]: 1.17, 4.18). Significantly higher mortality was found for CDI in IBD patients both short-term [OR: 3.84, 95% CI: 2.62, 5.61] and long-term [OR: 3.65, 95% CI: 1.58, 8.44] [60]. Therefore, it is important to reduce the incidence of CDI by avoiding unnecessary antibiotic treatments or choosing appropriate antibiotics, which can reduce the risk of mortality in IBD [61].

### 6.3. Antibiotic Risks

More than 90% of healthcare-associated *C. difficile* infections are linked to antibiotic therapy. The risk of infection increases with prolonged antibiotic use [62], but even a single dose can increase the risk. CDI may also appear up to several weeks after completion of antiobiotic therapy [63]. Different antibiotics have varying levels of association with *C. difficile* infection; they can be classified into three groups: high, moderate, and low risk. (Table 2) [64,65].

In dentistry, amoxicillin and ampicillin, as well as clindamycin, are commonly used as first- and second-line therapeutic and prophylactic antibiotics [16,66]. However, all three of these antibiotics have been shown to have a high risk of causing C. difficile infections in patients [15,64]. According to a meta-analysis by Brown Et Al. [67], clindamycin has been identified as having the highest risk of causing *C. difficile* infections among all antibiotics tested. Due to this fact, the American Dental Association has discontinued the recommendation of clindamycin in preoperative prophylaxis in dentistry [68]. Actis Et Al. posit the hypothesis that non-steroidal anti-inflammatory drugs (NSAIDs) and antibiotics (specifically those of the macrolide structure) have the capacity to induce intestinal and hepatic damage, thereby significantly enhancing co-morbidities in gastroenterological outpatients, with the result that cost-containment guidelines are no longer adhered to [69].

## 7. Management Strategies for Safe Antibiotic Use in Dentistry

### 7.1. Guidelines of Global Associations

The current recommendations of the; American Dental Association, American Association of Endodontists, National Institute for Health and Care Excellence, Polish Dental Association and National Programme To Protect Antibiotics Working Group, European Society of Endodontology regarding the use of antibiotics in dentistry do not provide specific guidance for antibiotic therapy in patients with IBD [16,70,71,72,73,74,75,76,77]. Only general recommendations for non-immunocompromised patients are provided, which include patients with Crohn’s disease and ulcerative colitis. However, due to the nature of these diseases, individual recommendations should be considered for them. Additionally, such recommendations have not been developed by the European Crohn’s and Colitis Organisation [76].

### 7.2. Metronidazole

One potential solution worth examining is the use of metronidazole, as it have the advantage of a lower risk of CDI compared to first- and second-line antibiotics in the dental treatment of patients with IBD.

Metronidazole has bactericidal activity and targets anaerobic microorganisms by inhibiting nucleic acid synthesis. It also has antiprotozoal activity and does not disrupt the protective aerobic microbiota [78,79,80].

It is commonly used in perioperative prophylaxis in procedures involving the large intestine and some procedures with access through the oral mucosa. The therapeutic properties of metronidazole in the treatment of exacerbations of CD and in maintaining remission are well-known [81,82]. Metronidazole is not an antibiotic, but a chemotherapeutic agent that belongs to the low-risk group for causing CDI [15]. It is also used as first-line treatment for *C. difficile* infection, although current guidelines indicate that vancomycin is more effective than metronidazole in IBD [58,76].

A study conducted by Ingham et al. to investigated the efficacy of metronidazole in the treatment of acute dental infections. This investigation compared the effectiveness of metronidazole with that of parenteral penicillin in a controlled trial. The study revealed that all 37 patients exhibited a positive response, suggesting that metronidazole is comparable in effectiveness to parenteral penicillin. A further 24 patients treated with metronidazole also exhibited satisfactory responses [83]. A meta-analysis was conducted by Raghavan et al. [84], which concluded with the recommendation of the pre- and post-operative use of metronidazole in patients undergoing third molar surgery to reduce the incidence of dry socket.

A single dose of metronidazole before surgery has been shown to be more effective for *C. difficile* prophylaxis than multiple doses of metronidazole plus cefuroxime [85]. In contrast to amoxicillin, cefotaxime, and vancomycin, metronidazoledid not exert a notable influence on the composition or diversity of the gut microbiota. This is presumably due to the fact that is readily absorbed in the small intestine, thereby limiting its impact on the lower gut, where the majority of microbiota are located [86].

### 7.3. Local Administration of Antibiotic Carriers

The administration of antibiotics Via advanced carrier systems has garnered mounting attention as a promising approach to mitigate systemic adverse effects and the risk of CDI, a matter of particular pertinence in patients with IBD [87]. Local drug delivery enables high concentrations of the active compound directly at the site of infection while significantly reducing systemic exposure. This concept is particularly advantageous in the field of dentistry, where infections are typically localised and confined to the soft or hard tissues of the oral cavity [88].

In recent studies, the potential of autologous platelet concentrates, such as platelet-rich fibrin (PRF) and concentrated growth factors (CGF), as innovative drug carriers has been investigated. The fibrin matrix, abundant in platelets and leukocytes, functions as a natural scaffold for the controlled release of drugs and enhances tissue regeneration through growth factors such as PDGF, TGF-β, and VEGF. A multitude of in vitro investigations have substantiated that PRF and CGF possess the capacity to effectively incorporate and steadily release antibiotics, including amoxicillin, metronidazole, and clindamycin, thereby preserving antimicrobial efficacy over an extended duration [89,90]. This prolonged, localised release profile has the potential to enhance infection control while minimising systemic antibiotic exposure, which is a pivotal benefit for IBD patients who are at risk of developing intestinal dysbiosis and CDI recurrence [91].

Recent findings indicate that PRF and CGF mixed with metronidazole or fluconazole exhibit potent antimicrobial and antifungal effects against common oral pathogens such as *Streptococcus viridans*, *Enterococcus faecalis, Candida albicans*, and *Prevotella intermedia*. Notably, the biological properties of these autologous biomaterials remain unaltered by the incorporation of antimicrobial agents, thereby enabling concurrent tissue regeneration and infection prevention. This renders them particularly well-suited for utilisation in periodontal surgery, implantology, and regenerative endodontic procedures in patients diagnosed with IBD [89,92].

The potential of alternative carrier systems, including hydrogels, nanofibers, microspheres, and biodegradable membranes, to deliver antibiotics topically in the oral cavity has been a subject of investigation. However, autologous fibrin matrices have been demonstrated to exhibit superior biocompatibility, availability, and cost-effectiveness, with the added benefit of reducing the reliance on synthetic materials and preservatives that could potentially induce hypersensitivity or inflammatory responses [88,93,94,95,96,97].

For patients diagnosed with IBD, local administration of antibiotics may offer a safe and effective adjunctive treatment, with benefits that include minimising systemic antibiotic load, protecting gut microbiota, and thereby reducing the risk of CDI. Further clinical trials are warranted to standardise preparation protocols, determine optimal drug concentrations, and confirm long-term safety and efficacy in both immunocompromised and systemically burdened populations.

### 7.4. Microbiome-Supportive Adjuncts

Adjunctive therapies such as probiotics, prebiotics, and synbiotics may support microbiome stability during and after antibiotic use. Although widely discussed in gastroenterology, their application in dentistry requires clear, evidence-based context. In patients with inflammatory bowel disease, these adjuncts may help reduce dysbiosis, mitigate antibiotic-associated gastrointestinal symptoms, and potentially lower the risk of *Clostridioides difficile* infection. However, their use must be carefully tailored to dental procedures and patient immunological status [98].

Probiotics, defined as live microorganisms that confer a health benefit to the host when administered in adequate amounts, have been the most widely studied biotics in this context. A substantial body of research, evidenced by numerous randomised clinical trials, has demonstrated that specific probiotic strains, including *Lactobacillus rhamnosus* GG, *Bifidobacterium longum*, and *Saccharomyces boulardii*, have the potential to reduce the incidence of antibiotic-associated diarrhoea and CDI in both healthy and immunocompromised populations. In patients diagnosed with IBD, the utilisation of probiotics has been a subject of investigation for its potential to contribute to the maintenance of remission and the reduction in disease activity. Studies have yielded more encouraging outcomes in UC than in CD. The multi-strain probiotic VSL#3 appears to provide the most consistent clinical benefit, although not all studies confirm its efficacy. Multi-strain formulations have demonstrated a greater potential for efficacy in comparison to single-strain probiotics [98,99,100].

In circumstances where systemic antibiotics are deemed necessary within the context of dental practice, the judicious administration of probiotic strains may contribute to the maintenance of gut microbiota stability, thereby mitigating the risk of developing antibiotic-associated diarrhoea in patients diagnosed with IBD. Evidence-supported strains include Saccharomyces boulardii, which has demonstrated benefit in preventing antibiotic-associated diarrhoea and reducing recurrence of C. difficile infection, as well as several Lactobacillus and Bifidobacterium species commonly used in clinical trials, such as *Lactobacillus rhamnosus GG*, *L. plantarum*, *L. acidophilus*, *L. casei*, *Bifidobacterium longum*, and *B. breve*. These strains may be considered as adjuncts during or immediately after antibiotic therapy, particularly in IBD patients with a history of dysbiosis or gastrointestinal sensitivity [101,102,103]. However, it is imperative to note that the utilisation of probiotics must be tailored to the individual, as their efficacy is not universally applicable, particularly in cases of immunosuppression. Contraindications for this treatment include severe immunosuppression, high-dose corticosteroid therapy, neutropenia, the presence of central venous catheters, critical illness, and severe active colitis with mucosal barrier disruption. In such cases, the risk of probiotic-related bacteremia or fungemia, particularly with *S. boulardii*, is considered to exceed the potential benefits. For patients receiving biologics or thiopurines, probiotics may be used cautiously and ideally in consultation with the patient’s gastroenterologist. Consequently, while certain strains may offer beneficial effects during dental antibiotic therapy, their use in IBD must be meticulously tailored to the patient’s immune status and overall clinical condition [100,104].

Prebiotics, such as inulin, fructooligosaccharides, and galactooligosaccharides, are non-digestible substrates that selectively stimulate the growth of beneficial bacteria like Bifidobacterium and Lactobacillus. While prebiotics are generally considered safe, their clinical efficacy in IBD remains unproven. The existing controlled trials are limited in scope and underpowered, demonstrating inconsistent or minimal benefits in modulating disease activity. While not inherently detrimental, prebiotics have been observed to induce mild gastrointestinal adverse effects, including bloating or discomfort. However, the administration of prebiotics during the early stages of life may have a significant impact on the development of the gut microbiome, which could subsequently influence an individual’s susceptibility to or protection from IBD in later life [100,105].

Synbiotics, a combination of probiotics and prebiotics, seek to enhance microbial balance in a synergistic manner. Preliminary evidence suggests that synbiotics may improve mucosal healing, reduce inflammation, and enhance gastrointestinal tolerance during antibiotic therapy. However, given the variability observed in probiotic–prebiotic combinations and dosages, it is premature to draw definitive conclusions regarding their efficacy [106,107].

Recent research has introduced paraprobiotics and postbiotics as promising alternatives. Paraprobiotics—non-viable microbial cells or their components—offer several advantages, including safety in immunocompromised individuals, absence of risk for bacterial translocation or antibiotic resistance gene transfer, and greater stability in production and storage. In a similar vein, postbiotics, comprising microbial metabolites such as short-chain fatty acids, have exhibited promising benefits on epithelial integrity and inflammation modulation. However, it should be noted that the current state of research is preliminary and heterogeneous [108,109].

## 8. Discussion

### 8.1. General Interpretation

Notwithstanding the rising prevalence of individuals afflicted with IBD who are concurrently afflicted with dental ailments, there persists an absence of formal guidelines to assist dental practitioners in the judicious selection of antibiotic therapy for this demographic. This discrepancy is of clinical significance, as numerous conventional dental antibiotics carry a high risk of *Clostridioides difficile* infection and gut microbiota disruption. Within this context, metronidazole offers significant potential due to its low CDI risk and robust anaerobic coverage; however, its limitations include its ineffectiveness against aerobic pathogens, the necessity for combination therapy in numerous odontogenic infections, and safety concerns such as alcohol interactions and neuropathy.

In order to further minimise systemic antibiotic exposure, local antimicrobial delivery systems, including PRF and CGF, represent promising adjuncts. These approaches have the potential to reduce the necessity for broad-spectrum systemic antibiotics by enhancing localised infection control and promoting tissue healing.

Furthermore, probiotics have been shown to provide a supportive benefit for selected IBD patients during or after systemic antibiotic therapy, particularly in terms of preventing antibiotic-associated gastrointestinal disturbances. However, the utilisation of these agents is not without caveats; the efficacy of these agents varies according to the specific strain of the pathogen, their benefits are largely supplementary rather than therapeutic, and their use may be contraindicated in individuals with compromised immune systems due to the risk of translocation or fungemia.

Collectively, these findings underscore the necessity for individualised, microbiome-conscious antibiotic strategies in the dental management of IBD patients and emphasise the importance of developing formal guidelines to support clinical decision-making.

### 8.2. Strengths

This review addresses a significant yet understudied clinical intersection: the safe utilisation of systemic antibiotics in the dental management of patients with inflammatory bowel disease. A key strength of the study is the comprehensive, multi-database search strategy, which captured a wide spectrum of medical, dental, microbiological, and pharmacological literature relevant to antibiotic decision-making in this high-risk population. The review also incorporates emerging topics, such as the potential role of local delivery systems (PRF, CGF) and microbiome-supportive adjuncts (probiotics), which are seldom discussed in dentistry despite growing clinical relevance. Finally, the review emphasises practical applicability, synthesising available evidence into clear clinical considerations that can support dentists in contexts where formal guidelines are lacking.

### 8.3. Limitations

It is important to note that this review is subject to several significant limitations. Firstly, there is a notable absence of studies specifically evaluating antibiotic therapy for IBD patients in the dental office, which restricts the ability to draw strong, evidence-based conclusions tailored to this population. The majority of available data are extrapolated from general medical or gastroenterological contexts, rather than from dental clinical trials involving patients with IBD.

Secondly, although PRF and CGF show promise as local antimicrobial carriers, the majority of evidence supporting their antibacterial activity and drug-delivery potential comes from in vitro studies. This limits the reliability and direct clinical applicability of these findings. In order to confirm the effectiveness and safety of the aforementioned treatment in a dental context, it is necessary to conduct high-quality in vivo or clinical studies.

In conclusion, due to the breadth and heterogeneity of the topic, it was only possible to synthesise the included literature in a narrative manner. The heterogeneity of the studies, in terms of their design, methodology and outcomes, precluded the application of standardised risk-of-bias tools or quantitative synthesis (e.g., meta-analysis). Consequently, the conclusions derived from this study should be interpreted with a degree of caution, taking into account the inherent limitations of the methodology employed in narrative reviews.

### 8.4. Future Directions

It is recommended that future research endeavours seek to address the significant evidence gaps that have been identified in this review. The necessity for clinical studies specifically involving IBD patients in dental settings is evident, particularly in the context of evaluating the efficacy and safety of antibiotics, the incidence of CDI, and the outcomes of microbiome interventions. In order to validate the antimicrobial and drug-delivery potential of PRF and CGF, it is essential to conduct high-quality in vivo and clinical trials, thus building upon preliminary in vitro findings. Furthermore, the potential of probiotics and synbiotics as adjunctive therapies during dental antibiotic treatment warrants exploration, with particular attention to strain-specific effects and safety in immunosuppressed individuals. Ultimately, multidisciplinary collaboration between dentistry, gastroenterology, microbiology, and pharmacology is essential to develop evidence-based, IBD-specific antibiotic guidelines that support safe and personalised dental care.

## 9. Conclusions

Patients suffering from inflammatory bowel disease present a unique set of challenges in the context of dental antibiotic management, due to their elevated risk of Clostridioides difficile infection and vulnerability to microbiome disruption. Notwithstanding these concerns, there are currently no dedicated guidelines to support dentists in selecting safe and effective antibiotic regimens for this population. This review underscores the potential role of metronidazole as a lower-risk option in selected cases, while acknowledging its limitations—including insufficient aerobic coverage and safety considerations—and emphasising the need for judicious, case-specific use rather than universal preference.

The utilisation of emerging alternatives, such as local antimicrobial delivery systems (PRF, CGF), has the potential to reduce reliance on systemic antibiotics. However, the current body of evidence is predominantly confined to in vitro studies and necessitates clinical validation. The use of probiotics and synbiotics has been demonstrated to provide a supportive benefit in certain IBD patients undergoing systemic antibiotic treatment. However, it is imperative to emphasise that the utilisation of these substances must be tailored to the individual, taking into account the strain-specific efficacy and contraindications in immunosuppressed individuals.

In conclusion, the extant evidence supports the implementation of a more personalised and microbiome-conscious approach to antibiotic therapy in dentistry for patients suffering from inflammatory bowel disease (IBD). There are still significant knowledge gaps, and well-designed clinical studies are urgently needed to establish evidence-based protocols that balance antimicrobial effectiveness with patient safety.

## Figures and Tables

**Table 1:** Syntaxes for the various databases.

Database	Search Terms	Number of Results
PubMed	(“Dentistry” OR “Oral health” OR “Stomatology”) AND (“CDI” OR “Clostridium difficile” OR “Clostridioides difficile” OR “periprocedural antibiotic therapy” OR “antibiotic therapy”) AND (“IBD” OR “Crohn disease” OR “inflammatory bowel disease” OR “Ulcerative Colitis”).	440
Embase	(‘Dentistry’ OR ‘Oral Health’ OR ‘Stomatology’) AND (‘CDI’ OR ‘Clostridium difficile’ OR ‘Clostridioides difficile’ OR ‘Periprocedural Antibiotic Therapy’ OR ‘Antibiotic Therapy’) AND (‘IBD’ OR ‘Crohn Disease’ OR ‘Inflammatory Bowel Disease’ OR ‘Ulcerative Colitis’)	258
Scopus	(“Oral”) AND (“CDI” OR “Clostridium difficile” OR “Clostridioides difficile” OR “Periprocedural Antibiotic Therapy” OR “Antibiotic Therapy”) AND (“IBD” OR “Crohn Disease” OR “Inflammatory Bowel Disease” OR “Ulcerative Colitis”)	587
Google Scholar	“Dentistry” OR “Oral Health” OR “Stomatology” AND “CDI” OR “Clostridium difficile” OR “Clostridioides difficile” OR “Periprocedural Antibiotic Therapy” OR “Antibiotic Therapy” AND “IBD” OR “Crohn Disease” OR “Inflammatory Bowel Disease” OR “Ulcerative Colitis”	500 most accurate results were included in the review process

**Table 2:** Relationship between antibiotic group and risk of CDI.

High Risk	Moderate Risk	Low Risk
- Fluoroquinolones - Second generation cephalosporins - Third generation cephalosporins - Clindamycin - Ampicillin - Amoxicillin - Broad-spectrum penicillins with inhibitors (except ticarcillin/clavulanate and piperacillin/tazobactam	- Macrolides - Trimethoprim/Sulfamethoxazole - Other penicillins - Sulfonamides	- Aminoglycosides - Bacitracin - Metronidazole - Teicoplanin - Vancomycin - Rifampicin - Chloramphenicol - Tetracycline - Carbapenems - Daptomycin - Tigecycline
